# Verification of tangential breast treatment dose calculations in a commercial 3D treatment planning system

**DOI:** 10.1120/jacmp.v2i2.2616

**Published:** 2001-03-01

**Authors:** Christopher T. Baird, George Starkschall, H. Helen Liu, Thomas A. Buchholz, Kenneth R. Hogstrom

**Affiliations:** ^1^ Department of Radiation Physics The University of Texas M. D. Anderson Cancer Center 1515 Holcombe Boulevard Houston Texas 77030; ^2^ Department of Clinical Radiation Oncology The University of Texas M. D. Anderson Cancer Center 1515 Holcombe Boulevard Houston Texas 77030

**Keywords:** *x*‐ray dose algorithm verification, tangential breast irradiation, tissue compensators

## Abstract

The accuracy of the photon convolution/superposition dose algorithm employed in a commercial radiation treatment planning system was evaluated for conditions simulating tangential breast treatment. A breast phantom was fabricated from machineable wax and placed on the chest wall of an anthropomorphic phantom. Radiographic film was used to measure the dose distribution at the axial midplane of the breast phantom. Subsequently, thermoluminescent dosimeters (TLDs) were used to measure the dose at four points within the midplane to validate the accuracy of the film dosimetry. Film measurements were compared with calculations performed using the treatment planning system for four types of treatment: optimized wedged beams at 6 and 18 MV and two‐dimensional compensated beams at 6 and 18 MV. Both the film‐ and TLD‐measured doses had a precision of approximately 0.6%. The film‐measured doses were approximately 1.5% lower than the TLD‐measured doses, ranging from 0–3 % at 6 MV and 0.5–1 % at 18 MV. Such results placed a high level of confidence in the accuracy and precision of the film data. The measured and calculated doses agreed to within ±3% for both the film and TLD measurements throughout the midplane exclusive of areas not having charged particle equilibrium. Good agreement was not expected within these regions due to the limitations in both film dosimetry and the dose‐calculation algorithm. These results indicated that the treatment planning system calculates doses at the midplane with clinically acceptable accuracy in conditions simulating tangential breast treatment.

PACS number(s): 87.53.–j, 87.66.–a

## INTRODUCTION

A standard radiation treatment technique for early‐stage breast cancer consists of using opposed tangential photon beams to treat the intact breast. Traditionally, two‐dimensional (2D) treatment planning has been used to plan radiation treatments of breast cancer. In this process, the dose distribution is optimized on the axial plane containing the central axis of the beam. While this method delivers a uniform dose on the central‐axis plane in small‐ to medium‐breasted women, significant volumes of increased dose (hot spots) have been reported in large‐breasted women due to the rapidly changing contour of their breasts as well as the larger beam entry separation distance.^1^ ^,^ ^2^ This dose inhomogeneity may have a direct effect on the cosmetic outcome of the patient.^3^ However, optimizing the dose distribution using three‐dimensional (3D) treatment planning can minimize this problem. Specifically, wedges, compensators, and multiple beams can optimize the dose distribution by visualizing the extent of dose inhomogeneity in the off‐axis planes.^4^ ^,^ ^5^ For such solutions, it is important that the 3D treatment planning system be able to calculate the dose accurately.

The purpose of the present study was to evaluate the accuracy of a 3D treatment planning system (Pinnacle^3^; ADAC Laboratories, Milpitas, CA) under conditions simulating tangential breast treatment model. This 3D treatment planning system computes photon doses based on a convolution/superposition photon beam.^6^ ^,^ ^7^ The parameters used in the beam model were obtained in a previous study by fitting measured data from the individual treatment machines acquired with the beam at normal incidence on a water phantom.^8^ In that study it was shown that the beam model in the 3D treatment planning system can reproduce relative doses that match measured doses in a water phantom to within 0.5–1.0% along the central axis and 2% along off‐axis profiles. However, the geometry of the water phantom used in beam modeling does not reflect the geometry used in breast cancer patients, which may affect the accuracy of dose calculation.

Several other studies have been done to verify the accuracy of the convolution/superposition algorithm; however, none of these studies have accurately simulated tangential breast irradiation. In particular, Papanikolaou *et al*.^7^ calculated percent depth doses for 6‐MV and 18‐MV fields, finding agreement to within approximately 1% along the central axis. In another study, an extended phantom geometry with the beam at normal incidence was used to calculate portal dose images.^9^ Also, dosimetry was performed on an anthropomorphic phantom using an electronic portal imaging device (EPID) and film. Results of the study indicated that the calculated portal dose image was within 3% of the EPID measurements and 4% of the film measurements in the central portion of the field.

Additional studies have verified the accuracy of the convolution/superposition algorithm for wedged and oblique fields, configurations that more closely approximate tangential breast irradiation. For example, Lydon^10^ examined the accuracy of the algorithm for a number of field geometries. These measurements were performed using an ionization chamber in a water phantom and indicated agreement to within 2% for most open and wedged fields; for the oblique fields, the measured data were within 2% of the treatment planning system calculation.

Finally, another study compared calculations for a solid water phantom performed using the Pinnacle^3^ treatment planning system with the AAPM Task Group 23 (TG‐23) data set.^11^ In that study, calculations were performed under a number of different conditions, including using oblique fields and inhomogeneous phantoms. The data indicated agreement between the treatment planning system and the TG‐23 data set to within 2% for 96% of the test points.

Unlike in these previous studies, our objective was to verify the photon‐dose algorithm under conditions that precisely mimic those in tangential breast irradiation. The hypothesis for this research was that the convolution/superposition algorithm calculates the dose in the midplane perpendicular to the posterior field edge that agrees with the measurement at an accuracy of ±3%. This hypothesis was tested using either wedged beams or beams with compensators.

## METHODS

### Phantom construction and setup

**Figure 1:**
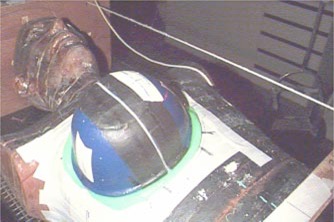
(Color) Positioning of the breast phantom on the anthropomorphic phantom. Crosshairs and laser lines are indicated on the phantoms. The markings were necessary on both phantoms to ensure that they were positioned correctly (i.e., the anthropomorphic phantom on the treatment couch and the breast phantom on the anthropomorphic phantom).

To simulate tangential breast anatomy, a right breast phantom (Fig. 1) was molded from modeling wax to fit a $1000-{\text{cm}}^{3}$ cup of a commercial treatment brassiere (Radiology Support Devices, Inc., Long Beach, CA). This modeling wax has a density of $0.920{\text{gcm}}^{-3}$, which is nearly equivalent to the density of breast adipose tissue.^12^ The posterior surface of the breast phantom was molded to fit the chest wall of the anthropomorphic phantom. Small air gaps between the chest‐wall surface of the breast phantom and that of the anthropomorphic phantom due to contraction of the wax as it cooled were filled with a thin layer of the malleable water‐based bolus TX‐151 (Oil Center Research Inc., Lafayette, LA). Next, the brassiere was used to immobilize the breast phantom on the anthropomorphic phantom. To ensure reproducible setup of the breast phantom on the anthropomorphic phantom, alignment markings were made on each of them to indicate their position relative to each other.

Treatment‐planning axial computed tomography (CT) images were obtained throughout the entire breast model using an image slice thickness and spacing of 3 mm. In addition, a transverse line registered the midtransverse plane of the breast phantom. Its intersection with a line in the cranial‐caudal direction created a fiducial mark, which was marked by thin solder wire and used to reference the medial‐lateral and cranial‐caudal displacement of the isocenter designated in the CT data set from the crosshair on the breast phantom surface. Determination of this displacement was necessary to recreate the positioning and geometry of the anthropomorphic phantom on the treatment couch. After the CT scan data were transferred to the treatment planning system, the isocenter and beam angles were determined.

**Figure 2:**
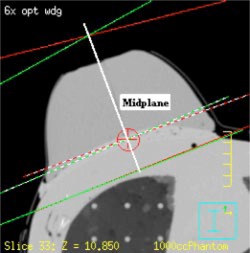
(Color) Midplane of the breast phantom. The white line passing through the phantom perpendicular to the posterior field edge represents the midplane in the transverse central‐axis plane.

**Figure 3:**
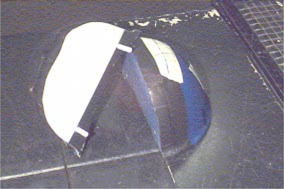
(Color) Breast phantom cut in half and showing the polyethylene filler and dowel. The film was cut to the size of the white area of the filler and placed between the filler and the opposite breast half. The positions of the isocentric positioning lasers are indicated by the crosshairs on the phantom.

The midplane of the breast phantom, which is the plane bisecting the angle formed by the lateral and medial central axes, is demarcated in Fig. 2. Because one of the aims of the present study was to measure the dose distribution at the midplane, it was necessary to split the breast phantom. To determine the location of the split, the breast phantom was placed in a linear accelerator (Clinac 2100‐C, Varian Medical Systems, Palo Alto, CA), and the setup was reproduced. The gantry was moved to the midplane angle, and the projection of the wire crosshairs in the cranial‐caudal direction, which designated the midplane, was marked on the breast phantom. The breast phantom was then removed from the anthropomorphic phantom and sawed into two pieces at the midplane; the surfaces of the pieces were smoothed using sandpaper. To compensate for the portion of the breast phantom that was lost in this process, a 2.5‐mm slab of polyethylene $\left(0.930{\text{gcm}}^{-3}\right)$ was placed between the two halves. Next, polyethylene dowels were used to align and connect the two halves of the breast phantom to the polyethylene filler. These dowels were positioned near the posterior surface of the breast, thereby creating a place for the film to rest on during irradiation (Fig. 3). Subsequently, another CT study of the phantom combination was performed using the same scan parameters.

### Treatment planning

The beam geometries were reproduced from the treatment plan developed using the first CT scan. Both field sizes were set to provide at least 1.5 cm of flash in the anterior, superior, and inferior directions, while the collimator angle was set to 0°. The gantry angles were set at slightly more than 180° apart, creating a coincident posterior beam edge for both fields. This border was selected at a depth that would provide coverage to the entire breast phantom. However, the anthropomorphic phantom did not provide a good anatomical representation of the lung position because its chest wall was excessively thick, so we did not attempt to include the lung in the treatment fields. In the wedged treatment plan, optimized dose distributions were created by combining an open beam with a wedged beam from both the lateral and medial fields. The open and wedged beams from each field were weighted relative to each other to produce isodose contours perpendicular to the common posterior field edge in the central‐axis plane. The lateral and medial fields were then weighted relative to each other to produce a homogeneous dose distribution in the plane containing the beam central axes. Off‐axis dose inhomogeneity was not considered in optimizing the plan.

In the compensated treatment plan, customized compensating filters were created using the compensator design algorithm in the Pinnacle^3^ system. In this algorithm, each compensating filter is designed to deliver a uniform dose to a plane perpendicular to the beam's central axis at a user‐specified depth. In the present study, this depth was 6 cm. Because the two beams were not opposed at a 180° angle, they did not share a common central axis, so the compensators had to be designed for two separate planes. However, because the beam central axis was slightly tilted, these planes were close enough to each other to provide good optimization. Nevertheless, the compensator filter design algorithm is limited in that the filters are designed for a given field independent of the other fields. In the present study, compensating filters were designed for both the lateral and medial fields. As before, these fields were weighted to deliver the most homogeneous dose.

Four plans were created using the treatment planning system: (1) a 6‐MV optimal wedge, (2) an 18‐MV optimal wedge, (3) a 6‐MV customized compensating filter, and (4) an 18‐MV customized compensating filter. In these plans, the lateral and medial field weighting was determined initially by visualizing the isodose contours in the transverse central‐axis plane. A dose‐volume histogram of the entire breast volume was used to make minor changes in this weighting.

### Compensator fabrication and quality assurance

Compensating filters were fabricated for the compensator thickness distribution calculated using the treatment planning system. The compensator information was exported to a milling machine (Autimo 2.5D; HEK, Lübeck, Germany), which was used to mill a negative mold of the compensator from Styrofoam (Soule Co., Tampa, FL). The depth of the negative mold was verified against the thickness array generated by the treatment planning system. Next, the styrofoam negative mold was filled with steel shot along with a small amount of beeswax for binding. The compensator was then mounted on a tray that was compatible with the wedge tray slot on the linear accelerator.

Quality assurance testing of the compensating filters was performed to ensure that they had been constructed correctly. First, the transmission factor of the compensator was measured using a Farmer‐type ionization chamber (PTW New York, Hicksville, NY) in a solid water phantom at the optimization‐plane depth along the central axis. Radiographic film (TVS; CEA America Corporation, Houston, TX) was used to measure the dose distributions in this plane. The film was placed perpendicular to the central axis of the beam in a solid water phantom at the geometry used for the transmission factor measurements. The transmission factors and dose distributions were compared with calculations made using the treatment planning system under identical conditions (patient replaced with a solid water phantom). For clinical use, the calculated and measured transmission factors on the central axis and the film doses off‐axis had to agree with each other to within ±2%.

### Verification of the dose plan

The dose distributions computed using the treatment planning system were verified by measuring both the dose distribution in the midplane of the breast phantom using radiographic film and point doses using thermoluminescent dosimeters (TLDs). A film and/or TLD was irradiated in a solid water phantom to a dose of 20 cGy at each session for calibration purposes. Pieces of film were cut in a darkroom in the shape of the cross section of the midplane within the breast phantom; they were then placed in the phantom to measure the planar dose distribution. The two halves of the breast phantom were secured together using photographic tape to eliminate potential light leaks.^13^ TVS film was chosen for dose‐distribution measurement because previous studies demonstrated that it is a suitable dosimeter for measuring high‐energy photon beams.^14^ ^,^ ^15^ The data obtained while measuring the sensitometric curve suggested that the film was linear up to a dose of approximately 30 cGy for both 6 and 18 MV. Therefore, a dose of 20 cGy was delivered to the prescription point within the phantom. Doses measured in the region not having charged particle equilibrium, the buildup region, and the region lateral to the breast surface (0.5 cm for 6 MV and 1.5 cm for 18 MV) were not evaluated due to the limitations of the convolution/superposition algorithm in areas of charged particle disequilibrium.^16^

**Figure 4:**
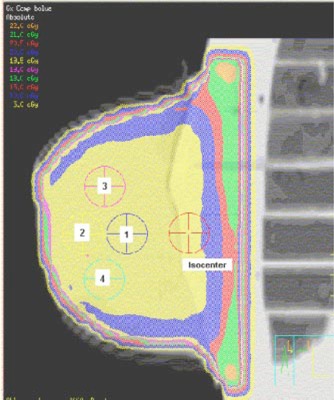
(Color) Locations of the TLD in the midplane of the breast phantom for the compensator plan. The midplane dose distribution in the 6‐MV 3D compensated 1000‐cm^3^ breast phantom is shown. The five points at which the midplane doses were measured using the TLD flat packs are labeled. Point 5 is at the isocenter.

TLD flat packs (TLD‐100; Harshaw Chemical Company, Solon, OH) were placed separately at four low‐dose‐gradient positions within the midplane to measure the point doses. The locations of the TLD for the compensator plans are shown in Fig. 4. Three separate measurements were performed for each point within the two plans. The mean and standard error of the mean were calculated using these measurements.

The measured and calculated doses in the midplane were compared using a data analysis and visualization software package (IDL Research Systems, Inc., Boulder, CO). The two dose matrices were aligned (translated and rotated) manually using an isodose line representing the phantom surface. For the calculated dose distribution, this line was the 5‐cGy isodose contour, as determined visually using the treatment planning system. However, for the measured dose distribution the 10‐cGy isodose contour occurred at the surface. This was determined when the film was scanned. Plots of the dose difference, as determined using the equation

$$\%\;\text{diff}=\frac{D_{calc}-D_{meas}}{D_{meas}}\times 100\%,$$

were generated using the IDL software to determine whether the calculated doses were within ±3% of the film measurements. The measured data were used as our standard because we were verifying the accuracy of the treatment planning system. In the above equation, $D_{\text{calc}}$ and $D_{\text{meas}}$ were the calculated and measured doses, respectively. Finally, doses measured at each point using the TLD were compared with doses calculated using the treatment planning system.

## RESULTS AND DISCUSSION

### Optimal wedge plan

**Figure 5:**
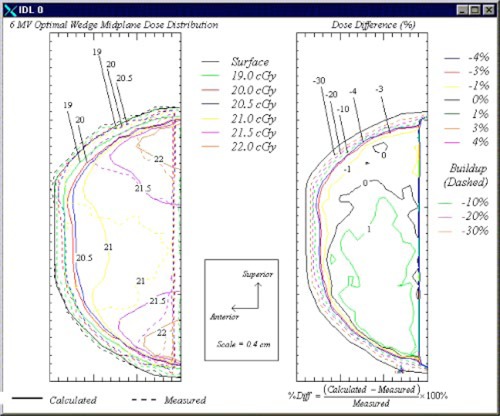
(Color) Sagittal‐plane isodose contours (left) and dose difference plot (right) for the 6‐MV optimal wedge plan. The dose difference plot reflects the difference between the dose measured using film and that calculated using the treatment planning system (scale=1 cm per small division).

**Figure 6:**
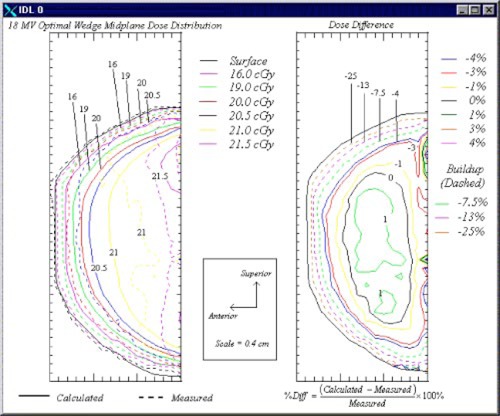
(Color) Sagittal‐plane isodose contours (left) and dose difference plot (right) for the 18‐MV optimal wedge plan.

Plots of the measured and calculated isodose contours in the sagittal breast midplane for the 6‐and 18‐MV beams are shown in Figs. 5 and 6, respectively. In each figure, the plot on the left displays both the calculated and measured dose distributions, while the plot on the right shows the percent differences between the two doses.

The plot of the dose distribution in Fig. 5 shows that hot spots on the order of 110% (22.0 cGy) of the prescribed dose were located in the superior and inferior regions of the breast phantom. The plot also shows that the wedges compensated for the dose in the anterior‐posterior direction. In this direction, the dose only varied from 20.5 to 21.0 cGy, or 2.5%. Additionally, the plot of the difference between the calculated and film‐measured doses is shown on the right side of the figure. The breast surface is indicated by the solid black line and is similar to the surface contour in the plot on the left side of the figure. This plot shows agreement to within ±3% throughout the midplane exclusive of the region in which charged particle equilibrium is not achieved. Throughout much of the midplane, the agreement is within ±1%. Also, the doses measured using film in the region in which charged particle equilibrium is not achieved were more than 30% higher than those calculated using the treatment planning system. Previous studies have reported an enhanced response of TVS film in the buildup region.^14^ ^,^ ^15^ In addition, we observed an energy dependence in the film exposed to 6‐MV photons that would correspond to an overresponse to the radiation at shallower depths in the breast phantom. This energy dependence was discovered by obtaining sensitometric curves at depths of 3.5 and 8 cm in a solid water phantom.

Results for the 18‐MV optimal wedge plan are shown in Fig. 6. As was the case with the 6‐MV beam, hot spots were visible superior and inferior to the central axis of the 18‐MV beams. However, these hot spots were smaller both in size and in magnitude (108% versus 110%) than those using the 6‐MV beams. This was a consequence of lesser attenuation for the 18‐MV beams. However, 18‐MV beams caused an increase in dose in the region in which charged particle equilibrium was not achieved inside the breast phantom because of the greater penetration of the secondary electrons.

The plot of the difference between the calculated and the measured doses is shown on the right side of the figure. The criterion of ±3% agreement between the calculation and measurement, exclusive of the region not achieving charged particle equilibrium, was met. The doses measured in this region using film were more than 25% higher than those calculated using the treatment planning system.

### 2D compensated plan

**Figure 7:**
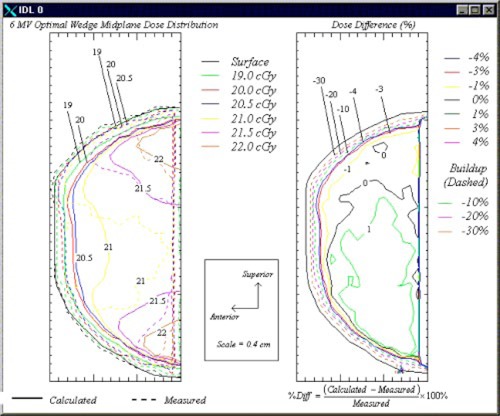
(Color) Isodose contours (left) and dose difference plot (right) in the 6‐MV compensator plan.

**Figure 8:**
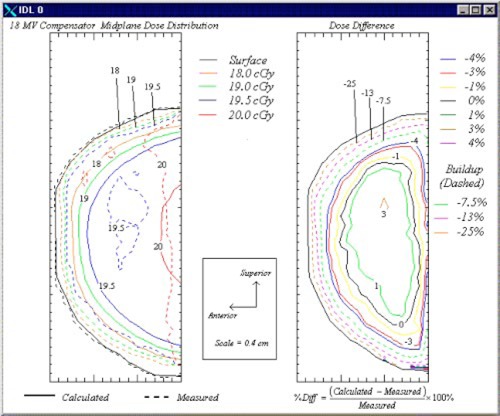
(Color) Isodose contours (left) and dose difference plot (right) in the 18‐MV compensator plan.

Because the purpose of 2D compensation is to improve the dose homogeneity within the breast, we also evaluated the accuracy of the treatment planning system when using customized compensating filters. The results of a comparison of the dose measured using film with the calculated isodose contours in the breast phantom midplane for 6‐ and 18‐MV beams are shown in Figs. 7 and 8, respectively. First, as expected, the use of 2D compensators eliminated the areas of increased dose in the superior and inferior regions of the breast, which were present in the 1D compensated, wedged plans. Agreement in the 6‐MV compensated plan was within ±3% exclusive of the region not achieving charged particle equilibrium except for small areas of the midplane in the superior and inferior regions near that region and the posterior edge of the film. In these areas, the calculated and measured doses differed by no more than 5%. Similar to the wedged plans, results in the region not achieving charged particle equilibrium indicated that the measured doses were greater than the calculated doses by at least 30% in the 6‐MV plan and 25% in the 18‐MV plan. Because the disagreement was similar to that in the wedged plan, it did not appear to be associated in any way with the fabrication of the compensating filters. In Fig. 8, the 18‐MV compensated plan shows agreement to within ±3% exclusive of the region not achieving charged particle equilibrium except for a minute area near the center of the midplane.

### Point doses

In Table I, TLD measurements at four dose points (as delineated in Fig. 4) are compared with the film‐measured doses and calculated doses. First, the precision of the measured data was extremely good: approximately 0.6% for both the TLD‐ and film‐measured data. Second, the film‐measured dose was slightly but statistically significantly lower than the TLD‐measured dose. At 6 MV, the film‐measured doses were 0–3 % lower than the TLD‐measured doses, while at 18 MV, the film‐measured doses were 0.5–1.0 % lower than the TLD‐measured doses.

**Table I:** Dose to the midplane of the $1000-{\text{cm}}^{3}$ breast phantom for (a) 6 MV and (b) 18 MV compensator plans. The mean dose and standard error were obtained from three separate measurements using either film or TLD flat packs. The beam‐on time for the measured data was based on a prescription of 20 cGy to the isocenter.

(a) 6 MV midplane dose						
Location	Calculated dose (cGy)	Film‐measured dose (cGy)	% Difference film (%)	TLD‐measured dose (cGy)	% Difference TLD (%)
1	19.65	19.4±0.1	–1.27	19.6±0.1	–0.25
2	19.26	19.5±0.1	–0.03	19.5±0.1	–0.31
3	19.62	19.4	0.2	–1.12	20.2±0.01	2.96
4	19.59	19.4±0.1	–0.97	19.9±0.1	1.58
(b) 18 MV midplane doses					
Location	Calculated dose (cGy)	Film‐measured dos (cGy)	% Difference film (%)	TLD‐measured dose (cGy)	% Difference TLD (%)
1	19.99	19.7±0.1	–1.45	20.0±0.3	0.05
2	19.88	19.6±0.1	–1.41	19.9±0.2	0.10
3	19.89	19.4±0.1	–2.41	19.6±0.1	–1.46
4	19.84	19.7±0.2	–0.71	19.8±0.1	–0.20

Comparison of the calculated dose with the TLD‐measured dose showed excellent agreement. At 6 MV, the calculated values at points 1, 2, and 4 were within 1% and at point 3 were within 2.7%; at 18 MV, the calculated values at points 1, 2, and 4 were within 0.3% and at point 3 were within 1.5%. These results showed the calculated values to be within 3% at all points. Comparison of the calculated dose with the film‐measured dose also showed good agreement. At 6 MV, the calculated values at points 1–4 were within 1.5%, while at 18 MV, the values were within 2.5%. The difference between the calculated and measured doses was greater than the precision of the data. Specifically, the calculated doses were systematically greater than the measured doses, averaging 0.9% for film‐measured doses and 1.0% for TLD‐measured doses at 6 MV and 1.0% for film‐measured doses and 0.4% for TLD‐measured doses at 18 MV. Also, at 6 MV, the calculated dose at the isocenter was approximately 3% less than the measured dose, which was possibly due to a difference in the isocenter position, while at 18 MV, the calculated and measured doses agreed to within 2%.

The results obtained using the phantom measurements indicated that the photon‐dose algorithm used in the Pinnacle^3^ treatment planning system is accurate to within ±3% in the sagittal midplane of the breast phantom, excluding the region in which charged particle equilibrium is not achieved.

## CONCLUSIONS

In the present study, we measured the radiation dose delivered to a breast phantom using parallel‐opposed tangential beams consisting of both optimal wedged and 2D‐compensated beams of 6‐ or 18‐MV *x* rays. Multiple sets of film data provided a measured dose distribution in the midplane of a breast phantom and indicated a precision of better than 1%. The accuracy of the film data in regions of charged particle equilibrium was evaluated at selected points by comparing them with data obtained using TLD, which is considered the more accurate dosimetry method. The film‐measured dose was lower than the TLD‐measured dose by an average of 1.6%. These data suggest that the measurements in the present study provided quality data for comparison with the dose algorithm used in the treatment planning system.

Our results showed that the convolution/superposition algorithm, as implemented in the ADAC Pinnacle^3^ treatment planning system, calculated the dose in the region of charged particle equilibrium in the midplane of the breast phantom at an accuracy rate of 3%. This allows practices to have a high degree of confidence in using the treatment planning software to plan optimized wedged and compensated beams in breast treatment planning. It should be noted that this comparison included dose errors that resulted from approximations in beam modeling, using the treatment planning system to calculate monitor units, modeling wedges or tissue compensators, and the algorithm modeling beam transport in irregularly shaped breasts.

We evaluated the accuracy of the dose‐calculation algorithm only in areas in which charged particle equilibrium existed within the midplane of the breast phantom. Areas in which charged particle equilibrium is not achieved, such as those at depths too shallow for forward scattering equilibrium or of grazing radiation lacking side‐scatter equilibrium, caused underestimation of the film response by as much as 30% near the breast phantom surface. These areas warrant further investigation to separate the dose differences due to algorithm deficiencies from those due to inaccuracy of the film measurements. This problem may be solvable using TLD measurement in these areas and calculation of the dose using Monte Carlo techniques.^17^ ^,^ ^18^

## ACKNOWLEDGMENTS

This research was supported in part by a sponsored research agreement with ADAC Laboratories, Milpitas, CA.

## References

[1] A. J. Neal , M. Torr , S. Helyer , and J. R. Yarnold , “Correlation of breast dose heterogeneity with breast size using 3D CT planning and dose‐volume histograms,” Radiother. Oncol. 34, 210–18 (1995). 7631027 10.1016/0167-8140(95)01521-h

[2] T. A. Buchholz , E. Gurgoze , W. S. Bice , and B. R. Prestige , “Dosimetric analysis of intact breast irradiation in off‐axis planes,” Int. J. Radiat. Oncol., Biol., Phys. 39, 261–267 (1997). 9300762 10.1016/s0360-3016(97)00292-7

[3] A. M. Moody , W. P. M. Mayles , J. M. Bliss , R. P. A'Hern , J. R. Owen , J. Regan , B. Broad , and J. R. Yarnold , “The influence of breast size on late radiation effects and association with radiotherapy dose inhomogeneities,” Radiother. Oncol. 33, 106–112 (1994). 7708953 10.1016/0167-8140(94)90063-9

[4] L. J. Solin , J. C. H. Chu , M. R. Sontag , L. Brewster , E. Cheng , K. Doppke , R. E. Drzymala , M. Hunt , R. Kuske , J. M. Manolis , B. McCormick , and J. E. Munzenrider , “Three‐dimensional photon treatment planning of the intact breast,” Int. J. Radiat. Oncol., Biol., Phys. 21, 193–203 (1991). 2032888 10.1016/0360-3016(91)90178-7

[5] C. Cheng , I. J. Das , and B. Stea , “The effect of the number of computed tomographic slices on dose distributions and evaluation of treatment planning systems for radiation therapy of the intact breast,” Int. J. Radiat. Oncol., Biol., Phys. 30, 183–195 (1994). 8083113 10.1016/0360-3016(94)90534-7

[6] T. R. Mackie , J. W. Scrimger , and J. J. Battista , “A convolution method of calculating dose for 15‐MV *x*‐rays,” Med. Phys. 12, 188–196 (1984). 10.1118/1.595774 4000075

[7] N. Papanikolaou , T. R. Mackie , C. Meger‐Wells , M. Gehring , and P. Reckwerdt , “Investigation of the convolution method for polyenergetic spectra,” Med. Phys. 20, 1327–1336 (1993). 8289713 10.1118/1.597154

[8] G. Starkschall , R. E. Steadham, Jr. , R. A. Popple , S. Ahmad , and I. I. Rosen , “Beam‐commissioning methodology for a three‐dimensional convolution/superposition photon dose algorithm,” J. Appl. Clin. Med. Phys. 1, 8–27 (2000). 11674815 10.1120/jacmp.v1i1.2651 PMC5726162

[9] T. R. McNutt , T. R. Mackie , P. Reckwerdt , N. Papanikolaou , and B. R. Paliwal , “Calculation of portal dose using the convolution/superposition method,” Med. Phys. 23, 527–535 (1996). 9157266 10.1118/1.597810

[10] J. M. Lydon , “Photon dose calculations in homogeneous media for a treatment planning system using a collapsed cone superposition convolution algorithm,” Phys. Med. Biol. 43, 1813–1822 (1998). 9651042 10.1088/0031-9155/43/6/031

[11] C. R. Ramsey , I. L. Cordrey , K. M. Spencer , and A. L. Oliver , “Dosimetric verification of two commercially available three‐dimensional treatment planning systems using the TG 23 test package,” Med. Phys. 26, 1188–1195 (1999). 10435518 10.1118/1.598614

[12] D. A. Low and K. R. Hogstrom , “Determination of the relative linear collision stopping power and linear scattering power of electron bolus material,” Phys. Med. Biol. 39, 1063–1068 (1994). 15551581 10.1088/0031-9155/39/6/012

[13] M. M. Ellen , K. R. Hogstrom , L. A. Miller , R. C. Erice , and T. A. Buchholz , “A Comparison of 18‐MV and 6‐MV treatment plans using 3D dose calculation with and without heterogeneity correction,” Med. Dosim. 24, 287–294 (1999). 10643738 10.1016/s0958-3947(99)00022-9

[14] C. Cheng and I. J. Das , “Dosimetry of high energy photon and electron beams with CEA films,” Med. Phys. 23, 1225–1232 (1996). 10.1118/1.597865 8839417

[15] P. Cadman , “Use of CEA TVS film for measuring high energy photon beam dose distributions,” Med. Phys. 25, 1435–1437 (1998). 9725130 10.1118/1.598316

[16] A. Ahnesjö , “Collapsed cone convolution of radiant energy for photon dose calculation in heterogeneous media,” Med. Phys. 16, 577–592 (1989). 2770632 10.1118/1.596360

[17] D. W. O. Rogers , B. Faddergon , G. Ding , C.‐M. Ma , J. We , and T. R. Mackie , “BEAM: A Monte Carlo code to simulate radiotherapy treatment units,” Med. Phys. 22, 503–524 (1995). 7643786 10.1118/1.597552

[18] C.‐M. Ma , E. Mok , A. Kapur , T. Pawlicki , D. Findley , S. Brain , K. Forster , and A. L. Boyer , “Clinical implementation of a Monte Carlo treatment planning system,” Med. Phys. 26, 2133–2143 (1999). 10535630 10.1118/1.598729

